# Mother and newborn skin-to-skin contact and timely initiation of breastfeeding in sub-Saharan Africa

**DOI:** 10.1371/journal.pone.0280053

**Published:** 2023-01-10

**Authors:** Richard Gyan Aboagye, Bright Opoku Ahinkorah, Abdul-Aziz Seidu, Stephen Kofi Anin, James Boadu Frimpong, John Elvis Hagan

**Affiliations:** 1 Department of Family and Community Health, Fred N. Binka School of Public Health, University of Health and Allied Sciences, Hohoe, Ghana; 2 School of Public Health, Faculty of Health, University of Technology Sydney, Sydney, Australia; 3 REMS Consult Limited, Sekondi-Takoradi, Western Region, Ghana; 4 College of Public Health, Medical and Veterinary Sciences, James Cook University, Townsville, Australia; 5 Centre For Gender and Advocacy, Takoradi Technical University, Takoradi, Ghana; 6 School of Public Health, Bielefeld University, Bielefeld, Germany; 7 Department of Industrial and Health Sciences, Faculty of Applied Sciences, Takoradi Technical University, Takoradi, Ghana; 8 Department of Health, Physical Education, and Recreation, University of Cape Coast, Cape Coast, Ghana; 9 Department of Kinesiology, New Mexico State University, Las Cruces, NM, United States of America; 10 Neurocognition and Action-Biomechanics-Research Group, Faculty of Psychology and Sport Sciences, Bielefeld University, Bielefeld, Germany; University of Salamanca, SPAIN

## Abstract

**Background:**

Mother and newborn skin-to-skin contact (SSC) plays a key role in breastfeeding practices of mothers. In this study, we examined the association between mother and newborn SSC and timely initiation of breastfeeding in sub-Saharan Africa (SSA).

**Methods:**

This cross-sectional study utilized nationally representative data from the Demographic and Health Surveys of 17 countries in SSA from 2015 to 2020. Multilevel binary logistic regression analysis was performed to examine the association between mother and newborn SSC and timely initiation of breastfeeding. The results are presented using adjusted odds ratios (aOR), with 95% confidence interval (CI).

**Results:**

The pooled prevalences of mother and newborn SSC and timely initiation of breastfeeding were 45.68% (95% CI = 34.12–57.23) and 62.89% (95% CI = 55.67–70.11), respectively. Mothers who practiced newborn SSC were more likely to practice timely initiation of breastfeeding compared to those who did not practice SSC [aOR = 1.68, 95% CI = 1.58, 1.78] and this persisted after controlling for all the covariates [aOR = 1.38, 95% CI = 1.29, 1.47]. At the country level, mother and newborn SSC increased the odds of timely initiation of breastfeeding in Angola [aOR = 1.99, 95% CI = 1.44, 2.76], Cameroon [aOR = 1.43, 95% CI = 1.02, 1.99], Ethiopia [aOR = 1.62, 95% CI = 1.16, 2.28], Guinea [aOR = 1.69, 95% CI = 1.10, 2.60], Liberia [aOR = 2.03, 95% CI = 1.33, 3.12], Malawi [aOR = 1.47, 95% CI = 1.02, 2.12], Mali [aOR = 1.42, 95% CI = 1.10, 1.84], Sierra Leone [aOR = 1.87, 95% CI = 1.23, 2.83], South Africa [aOR = 2.59, 95% CI = 1.41, 4.76], Tanzania [aOR = 1.60, 95% CI = 1.27, 2.01], Uganda [aOR = 1.43, 95% CI = 1.02, 1.99], Zambia [aOR = 1.86, 95% CI = 1.50, 2.30], and Zimbabwe [aOR = 1.65, 95% CI = 1.24, 2.21].

**Conclusion:**

The prevalence of SCC was relatively low but timely initiation of breastfeeding was high. Mother and newborn SSC is a strong predictor of timely initiation of breastfeeding in SSA. To enhance timely initiation of breastfeeding after birth, this study recommends that more child and maternal healthcare interventions focused on improving mother and newborn SSC should be implemented.

## Introduction

Malnutrition-related causes of neonatal morbidity, mortality, and impaired child growth coupled with the consequential effects on socio-economic development remains a major public health concern especially in developing countries [1–6]. The risk of under five mortality when initiation of breastfeeding is delayed in sub-Saharan Africa (SSA) was estimated to be three-fold higher compared to any kind of infant feeding as 55.3% of under-five mortality is attributable to delayed initiation of breastfeeding [1].

The importance of breastfeeding for both the mother and the baby during the period of breastfeeding and in the future have been well documented in literature [7–9]. For example, Alimoradi et al. [7] indicated that breast milk is the best food for children in the first two years of their life and replacing it with any other food is not recommended. Studies have shown that breastfeeding increases the child’s innate nutritional behavior including searching and sucking, which is critical for the child’s growth and survival as the mother exclusively feeds them [8, 9]. Evidence suggests that breastfeeding protects infants from several diseases (e.g., diarrhea, malaria) and reduce the severity of disease symptoms [10–12]. It also reduces the risk of childhood obesity [13], type 1 diabetes [14, 15], asthma [16] and epilepsy [17]. On the part of mother, it reduces the risk of breast cancer [18–20].

Timely initiation of breastfeeding (TIBF) is one of the core infants and young child feeding (IYCF) practices highly recommended by the World Health Organisation (WHO), United Nations Children’s Fund (UNICEF), and paediatric health experts to ensure optimal nutrition for the development, growth, and overall health of neonates [21]. TIBF also reduces neonatal and early infant mortality in addition to being predictive of optimal exclusive and/or continued breastfeeding [22, 23]. TIBF which is defined as breastfeeding newly born babies (neonates) within the first hour of birth [24], whether by the biological mother or a wet nurse, was reported to have a low pooled prevalence across thirty-two countries in SSA [25, 26].

Skin-to-skin contact (SSC) also known as Kangaroo Care or Kangaroo Mother Care refers to the practice of placing newborns without clothes (except maybe a diaper) on their mother’s bare chest, with warm coverings such as baby blankets or towels covering the newborn’s backside during the first few hours of childbirth and/or the postpartum period (typically the first 3 months of life) [27]. It is recommended that newborns are provided SSC given the many putative benefits such as mother-infant bonding facilitated by increased oxytocin (maternal reproductive hormone) production, enhanced breast-milk production, and breastfeeding success [28]. Breastmilk produced after delivery (parturition) within this sensitive period of the newborn’s life, is rich in colostrum, a thick complex biological yellowish fluid rich in nutrients, growth factors, and antibodies [29]. SSC also provides other physiological and psychological needs of the mother-infant dyad such as good sleep, joy, stable heart rates, optimal oxygen saturation levels, stable blood glucose levels, normal breathing patterns, enhanced temperature regulation, energy conservation for infant growth and weight gain, populating the infant with beneficial microbiota from its mother’s skin, and decreased infant crying [28, 30, 31]. Compared to newborns who were not given SSC, babies who were given SSC were found to have shown more of such facilitative neonatal health indicators and/or outcomes [30].

Several studies have examined the prevalence of TIBF and the risk factors associated with it in some individual and regional blocs of developing countries, especially in SSA [25, 32–37]. There is however the need to provide more empirical evidence of the postulated association between SSC and TIBF [38], particularly within the context of SSA, given the wide array of context-specific risk factors and/or covariates of TIBF identified from previous studies in SSA. This study sought to determine the country-specific and pooled prevalence of TIBF in SSA. The study also examined the association between mother and newborn SSC and TIBF in SSA.

## Materials and methods

### Data source and study design

A cross-sectional analysis of data from the most recent Demographic and Health Survey (DHS) conducted from 2015 to 2020 in SSA was performed. A total of 17 countries with datasets conducted within this period were included in the study. We included only 17 countries with the key explanatory variable (SSC). This variable was introduced in the DHS from 2015 upwards. We pooled the data from the kid’s recode (KR File). DHS is a comparable nationally representative survey conducted in over 85 low and middle-income countries (LMICs) worldwide [39]. The DHS employed a cross-sectional design, adopting a two-stage cluster sampling technique to recruit respondents for the survey. In the DHS, the first stage of sampling consisted of compiling a list of primary sampling units (PSUs) or enumeration areas (EAs) that covered the entire country, which was obtained from the most recent national census. The EAs were further subdivided into standardized segments of between 100–500 households each. Later, a random sample of a predetermined segment is chosen with a probability proportional to the size of the EA. In the second stage, households were systematically selected from a list of previously enumerated households in each selected EA segment and those who were usual residents of selected households or visitors who slept in the households on the night before the survey are interviewed. A structured questionnaire was used to collect data from the respondents on health indicators such as infant and young child feeding practices [39]. All DHS questionnaires are standardized instruments with high levels of reliability. In this study, we included women who had given birth 2 years preceding the survey. Table 1 shows the description of the study sample. A total sample size of 45,096 was used in the final analysis. This manuscript was drafted following the Strengthening Reporting of Observational Studies in Epidemiology (STROBE) guidelines [40]. The dataset is freely available for download at https://dhsprogram.com/data/available-datasets.cfm.

**Table 1:** Description of the study sample.

Countries	Year of survey	Weighted N	Weighted %
1. Angola	2015–16	2626	5.8
2. Benin	2017–18	5212	11.6
3. Burundi	2016–17	2259	5.0
4. Cameroon	2018	1887	4.2
5. Ethiopia	2016	4213	9.3
6. Gambia	2019–20	1595	3.5
7. Guinea	2018	1375	3.0
8. Liberia	2019–20	1030	2.3
9. Mali	2018	3901	8.7
10. Malawi	2015–16	2194	4.9
11. Nigeria	2018	4899	10.9
12. Sierra Leone	2019	1760	3.9
13. Tanzania	2015–16	4074	9.0
14. Uganda	2016	1869	4.1
15. South Africa	2016	509	1.1
16. Zambia	2018	3810	8.5
17. Zimbabwe	2015	1883	4.2
**All countries**	**2015–2020**	**45096**	**100.0**

### Variables

#### Outcome variable

TIBF was the outcome variable. To assess this variable, the women were asked the question “How long after birth did you first put [NAME] to the breast?” The response options were ‘immediately, within first hour, hours, and days. We coded the response options into a binary form such that those whose response options were ‘immediately and within the first hour were coded as “1 = Yes”, which implied that the woman performed TIBF. Those that responded ‘hours and days’ were coded as “0 = No”, signifying late initiation to breastfeeding. We coded the outcome variable with reference to literature that used the DHS datasets [25, 41–44].

#### Explanatory variables

The key explanatory variable in this study was the practice of mother and newborn SSC. This variable was derived from the question “Was child put on mother’s chest and bare skin after birth?” The responses to this question were “no”, “put on chest, touching bare skin”, “put on chest, no touching of bare skin”, “put on chest, don’t know/missing on touching on bare skin”, and “don’t know”. We dichotomised the responses into ‘Yes [mother and newborn SSC]’ for those that responded, “put on chest, touching bare skin”. The remaining responses were coded as ‘No [no mother and newborn SSC]. We based the categorisation and coding on previous studies [45, 46].

A total of 17 variables were included in the study as covariates. These variables were further grouped into individual-level (child and maternal characteristics) and contextual-level variables. The individual-level variables consisted of sex of child, birth order, birth weight, type of delivery, type of birth, mother’s age, educational level, marital status, current working status, antenatal care attendance, place of delivery, frequency of listening to radio, frequency of reading newspaper or magazine, and frequency of watching television. Wealth index, place of residence, and geographical subregions were the contextual-level variables. All the covariates were selected based on their significant association with breastfeeding [25, 41–44, 47] as well as their availability in the DHS dataset.

### Statistical analyses

The analysis was performed using Stata software version 16.0 (Stata Corporation, College Station, TX, USA). Percentages were used to summarise the prevalence of TIBF and mother and newborn SSC. Cross-tabulation was performed to determine the distribution of TIBF across the explanatory variables. Pearson chi-square test of independence was also used to examine the variables significantly associated with TIBF. Later, the best selection method was used to select the covariates to include in the multilevel regression analysis [48, 49]. With this method, we used the Stata command ‘gvselect’ to help estimate the combined set of variables to use. The log-likelihood, Akaike’s information criterion (AIC), and Bayesian information criterion (BIC) were the outcomes of the best selection method analysis. We chose the set of variables with the lowest AIC value, which according to the literature, is the best and preferred set of variables to include in a regression model [48, 49]. We employed multilevel binary regression analysis to examine the association between mother and newborn SSC and TIBF using five model (Model O-IV). We fitted the first model (Model O) to examine the variance in TIBF attributable to the clustering of primary sampling unit (PSU). In the Model I, we included only the mother and newborn SSC and TIBF. Model II was built to include TIBF and mother and newborn SSC, controlling for the individual-level covariates. Model III was also fitted to contain TIBF and mother and newborn SSC, controlling for the contextual-level covariates. The last model (Model IV) was fitted to contain TIBF and mother and newborn SSC, adjusting for both the individual-level and contextual-level covariates. The results were presented using crude odds ratio (cOR) and adjusted odds ratio (aOR), with their respective 95% confidence interval (CIs). Statistical significance was set at p<0.05. Model fitness and comparison were checked using the AIC, where the model with the least AIC being the best-fitted model. In all the analyses performed, we applied the sampling weight (v005/1,000,000), clustering, and stratification variables provided by DHS to account for the complex survey design.

### Ethical consideration

Ethical approval was not sought for this study since the dataset is freely available in the public domain. However, permission to use the data for publication was sought from the MEASURE DHS. We adhered to the ethical guidelines concerning the use of secondary dataset in publication. The detailed ethical guidelines and standards can be found at http://goo.gl/ny8T6X.

## Results

### Prevalence of mother and newborn skin-to-skin contact and timely initiation of breastfeeding in sub-Saharan Africa

**Fig 1:**
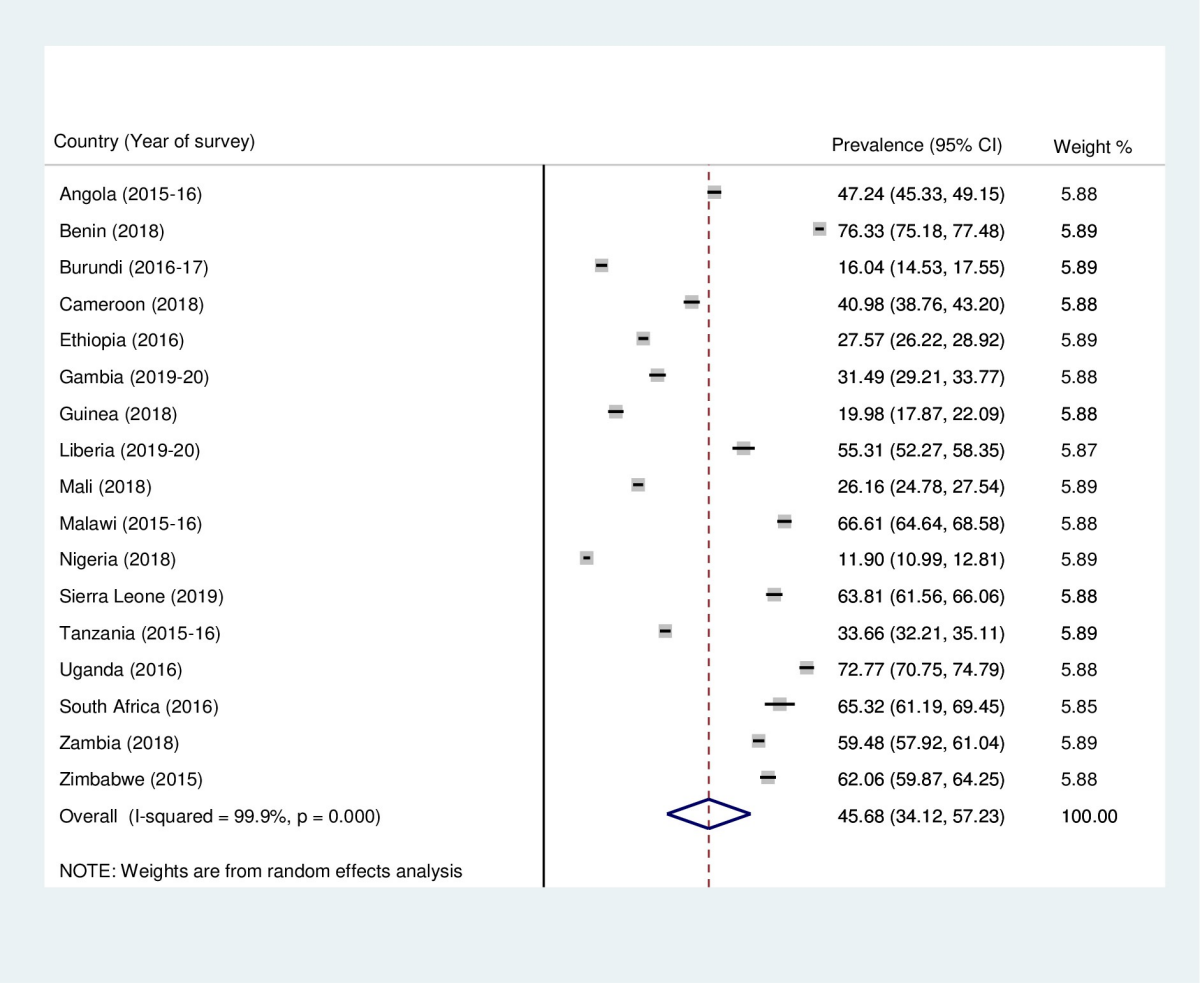
Forest plot showing the prevalence of mother and newborn skin-to-skin contact in sub-Saharan Africa.

**Fig 2:**
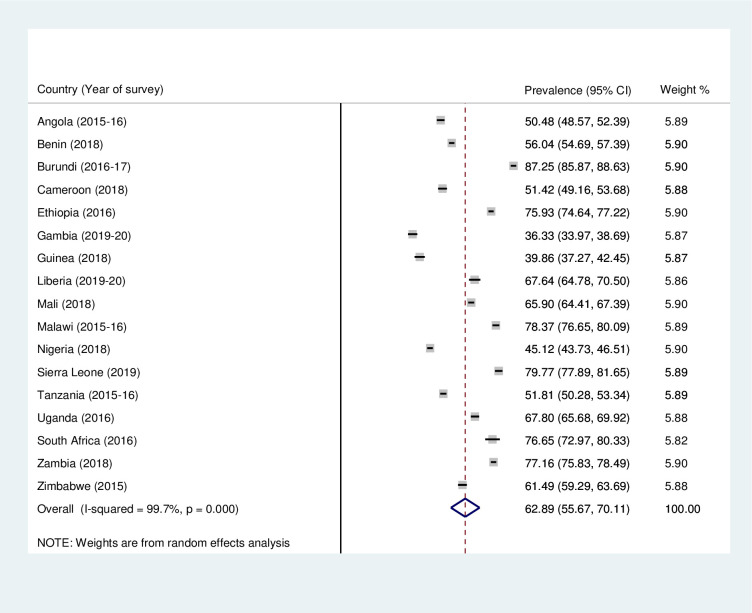
Forest plot showing the prevalence of timely initiation of breastfeeding in sub-Saharan Africa.

Figs 1 and 2 present the country-specific and pooled prevalences of mother and newborn SSC and TIBF in SSA. The results showed that the pooled prevalence of SSC and TIBF in SSA were 45.68% (95% CI = 34.12–57.23) and 62.89% (95% CI = 55.67–70.11), respectively. With the country-specific prevalence, the study found that while Nigeria recorded the least prevalence of SSC (11.90%; 95% CI = 10.99–12.81), Benin recorded the highest (76.33%; 95% CI = 75.18–77.48). Moreover, the prevalence of TIBF ranged from 36.33% (95% CI = 33.97–88.69) in Gambia to 87.25% (95% CI = 85.87–88.63) in Burundi.

### Association between timely initiation of breastfeeding and explanatory variables

Table 2 outlines the results of the association between TIBF and the explanatory variables. The study found that mother and newborn SSC (*p* < 0.001), birth order (*p* < 0.001), type of delivery (*p* < 0.001), maternal educational level (*p* < 0.001), marital status (*p* = 0.027), current working status (*p* < 0.001), place of delivery (*p* < 0.001), frequency of watching television (*p* < 0.001), frequency of listening to radio (*p* < 0.001), frequency of reading newspaper/magazine (*p* = 0.006), geographical sub-regions (*p* < 0.001) were significantly associated with TIBF.

**Table 2:** Distribution of timely initiation to breastfeeding across the explanatory variables.

Variables	Weighted N	Weighted %	Timely initiation of breastfeeding		
			No (%)	Yes (%)	p-value
**Mother and newborn skin-to-skin contact**					<0.001
No	25547	56.7	43.1	56.9	
Yes	19549	43.4	31.2	68.8	
**Sex of child**					0.136
Male	22777	50.5	38.4	61.6	
Female	22319	49.5	37.5	62.5	
**Birth order**					<0.001
First	9990	22.1	41.0	59.0	
Second	8610	19.1	35.9	64.1	
Third	7301	16.2	36.9	63.1	
Fourth	5754	12.8	37.1	62.9	
Fifth or more	13440	29.8	38.0	62.0	
**Birth weight**					0.221
Normal (≥2.5kg)	42732	94.8	37.9	62.1	
Low birthweight (<2.5kg)	2364	5.2	39.4	60.6	
**Type of delivery**					<0.001
Vaginal	42898	95.1	36.4	63.6	
Caesarean section	2198	4.9	68.3	31.7	
**Type of birth**					
Single	44370	98.4	37.8	62.2	
Multiple	726	1.6	50.0	50.0	
**Mother’s age (years)**					0.260
15–19	4681	10.4	39.6	60.4	
20–24	11380	25.2	38.3	61.7	
25–29	13038	26.7	37.5	62.5	
30–34	8611	19.1	37.2	62.8	
35–39	5717	12.7	37.5	62.5	
40–44	2197	4.8	38.5	61.5	
45–49	471	1.1	41.0	59.0	
**Maternal educational level**					<0.001
No education	17251	38.3	38.8	61.2	
Primary	14843	32.9	35.4	64.6	
Secondary	11424	25.3	39.2	60.8	
Higher	1578	3.5	43.3	56.7	
**Marital status**					0.027
Never in union	3274	7.3	36.6	63.4	
Married	33186	73.5	37.7	62.3	
Cohabiting	6428	0.8	40.2	59.8	
Widowed	347	1.4	35.0	65.0	
Divorced	648	2.7	34.1	65.9	
Separated	1214	2.7	38.6	61.4	
**Current working status**					<0.001
No	17513	38.8	35.7	64.3	
Yes	27583	61.2	39.4	60.6	
**Antenatal care visits**					0.106
None	4978	11.0	39.5	60.5	
1–3	14513	32.2	38.4	61.6	
4 or more	25605	56.8	37.4	62.6	
**Place of delivery**					<0.001
Home	12754	28.3	45.2	54.8	
Health facility	31823	70.6	35.0	65.0	
Other	519	1.1	43.8	56.2	
**Frequency of watching television**					<0.001
Not at all	27838	61.7	35.5	64.5	
Less than once a week	6290	14.0	42.0	58.0	
At least once a week	10968	24.3	42.0	58.0	
**Frequency of listening to radio**					<0.001
Not at all	20922	46.4	35.6	64.4	
Less than once a week	9339	20.7	41.3	58.7	
At least once a week	14835	32.9	39.1	60.9	
**Frequency of reading newspaper/magazine**					0.006
Not at all	38609	85.6	37.6	62.4	
Less than once a week	4108	9.1	40.9	59.1	
At least once a week	2379	5.3	39.1	60.9	
**Wealth index**					0.133
Poorest	10141	22.5	39.1	60.9	
Poorer	9803	21.7	38.7	61.3	
Middle	9172	20.3	37.1	62.9	
Richer	8482	18.8	37.6	62.4	
Richest	7498	16.6	36.9	63.1	
**Place of residence**					
Urban	14507	32.2	42.0	58.0	
Rural	30589	67.8	36.0	64.0	
**Geographical subregions**					<0.001
Southern	6201	13.8	27.6	72.4	
Central	4513	10.0	49.1	50.9	
Eastern	14609	32.4	29.7	70.3	
Western	19773	43.8	44.7	55.3	

*P-values were generated from the chi-square test

### Association between mother and newborn skin-to-skin contact and timely initiation of breastfeeding

Table 3 presents the results of association between SSC and TIBF. In Model I, infants whose mothers practiced SSC were more likely to receive TIBF [aOR = 1.68, 95% CI = 1.59, 1.79] after controlling for the key explanatory variable and this persisted after adjusting for the individual-level factors in Model II but with a reduced effect [aOR = 1.36, 95% CI = 1.28, 1.45]. Moreover, after controlling for the contextual variables in Model III, infants whose mothers practiced SSC were more likely to receive TIBF [aOR = 1.68, 95% CI = 1.58, 1.78] and this persisted after controlling for all the covariates in Model IV but with a reduced effect [aOR = 1.38, 95% CI = 1.29, 1.47].

**Table 3:** Fixed and random effect analysis of association between mother and newborn skin to skin contact and timely initiation of breastfeeding.

Variables	Model O	Model I cOR [95% CI]	Model II aOR [95% CI]	Model III aOR [95% CI]	Model IV aOR [95% CI]
**Fixed-effect results**					
**Mother and newborn skin to skin contact**					
No		1.00	1.00	1.00	1.00
Yes		1.68*** [1.59,1.79]	1.36*** [1.28,1.45]	1.68*** [1.58,1.78]	1.38*** [1.29,1.47]
**Random effect results**					
PSU variance (95% CI)	0.132 [0.108, 0.161]	0.128 [0.104, 0.156]	0.132 [0.108, 0.162]	0.138 [0.114, 0.166]	0.141 [0.116, 0.171]
ICC	0.038	0.037	0.039	0.042	0.041
Wald chi-square	Reference	291.56***	1263.79***	867.44***	1862.83***
**Model fitness**					
Log-likelihood	-29701.517	-29374.6	-28556.10	-28665.30	-27893.15
AIC	59407.03	58755.2	57178.2	57352.61	55868.3
N	45096	45096	45096	45096	45096
Number of clusters	1357	1357	1357	1357	1357

Exponentiated coefficients; 95% confidence intervals in brackets; aOR = adjusted odds ratios; CI = Confidence Interval;

* *p* < 0.05,

** *p* < 0.01,

*** *p* < 0.001; 1 = Reference category; PSU = Primary Sampling Unit; ICC = Intra-Class Correlation; AIC = Akaike’s Information Criterion;

Model I = Adjusted for only the key explanatory variable;

Model II = Adjusted for the key explanatory variable and individual-level variables;

Model III = Adjusted for the key explanatory variable and contextual variables;

Model IV = Adjusted for the key explanatory variable all the covariates obtained from the best variable selection method.

### Association between mother and newborn skin-to-skin contact and timely initiation of breastfeeding segregated by country

Table 4 presents the association between SSC and TIBF after segregating the results by country. The results showed that the odds of infants receiving TIBF among mothers who practiced SSC was higher in Angola [aOR = 1.99, 95% CI = 1.44, 2.76]; Cameroon [aOR = 1.43, 95% CI = 1.02, 1.99]; Ethiopia [aOR = 1.62, 95% CI = 1.16, 2.28]; Guinea [aOR = 1.69, 95% CI = 1.10, 2.60]; Liberia [aOR = 2.03, 95% CI = 1.33, 3.12]; Mali [aOR = 1.42, 95% CI = 1.10, 1.84]; Malawi [aOR = 1.47, 95% CI = 1.02, 2.12]; Sierra Leone [aOR = 1.87, 95% CI = 1.23, 2.83]; Tanzania [aOR = 1.60, 95% CI = 1.27, 2.01]; Uganda [aOR = 1.43, 95% CI = 1.02, 1.99]; South Africa [aOR = 2.59, 95% CI = 1.41, 4.76]; Zambia [aOR = 1.86, 95% CI = 1.50, 2.30], and Zimbabwe [aOR = 1.65, 95% CI = 1.24, 2.21].

**Table 4:** Association between mother and newborn skin to skin contact and timely initiation of breastfeeding segregated by country.

Countries	cOR [95% CI]	aOR [95% CI]
1. Angola	2.05*** [1.52, 2.77]	1.99*** [1.44, 2.76]
2. Benin	1.03 [0.79, 1.34]	0.94 [0.71, 1.24]
3. Burundi	1.48 [0.96, 2.28]	1.29 [0.81, 2.06]
4. Cameroon	1.62** [1.22, 2.16]	1.43* [1.02, 1.99]
5. Ethiopia	1.71*** [1.30, 2.25]	1.62** [1.16, 2.28]
6. Gambia	1.11 [0.82, 1.50]	1.06 [0.78, 1.45]
7. Guinea	1.95** [1.29, 2.96]	1.69* [1.10, 2.60]
8. Liberia	2.37*** [1.55, 3.61]	2.03** [1.33, 3.12]
9. Mali	1.58*** [1.23, 2.02]	1.42** [1.10, 1.84]
10. Malawi	1.71** [1.21, 2.40]	1.47* [1.02, 2.12]
11. Nigeria	1.07 [0.82, 1.38]	0.94 [0.72, 1.24]
12. Sierra Leone	1.75** [1.18, 2.58]	1.87** [1.23, 2.83]
13. Tanzania	2.65*** [2.14, 3.28]	1.60*** [1.27, 2.01]
14. Uganda	2.01*** [1.52, 2.66]	1.43* [1.02, 1.99]
15. South Africa	2.86** [1.58, 5.17]	2.59** [1.41, 4.76]
16. Zambia	3.25*** [2.58, 4.10]	1.86*** [1.50, 2.30]
17. Zimbabwe	2.15*** [1.64, 2.81]	1.65** [1.24, 2.21]

Exponentiated coefficients; 95% confidence intervals in brackets; aOR = adjusted odds ratios; CI = Confidence Interval;

* *p* < 0.05,

** *p* < 0.01,

*** *p* < 0.001

## Discussion

This study sought to determine the country-specific and pooled prevalence of TIBF in SSA. The study also examined the association between mother and newborn SSC and TIBF in SSA. The results showed that the pooled prevalence of SSC and TIBF in SSA were 45.68% and 62.89%, respectively. With the country-specific prevalence, the study found that while Nigeria recorded the least (11.90%) prevalence of SSC, Benin recorded the highest (76.33%). Another recent study [45] also confirmed the low prevalence of SSC in Nigeria. It could be that Nigerian women do not see the importance of SSC as a means of warming their babies after delivery, making them less likely to adopt SSC [50]. Alternatively, Nigerian women may not be aware of the benefits of practicing SSC, hence, their less likelihood to embrace and practice SSC [28]. It is also possible the existence of certain cultural practices and beliefs discourage women in Nigeria from practicing SSC [51, 52]. Further, inadequate knowledge on SSC on the part of health personnel, inadequate health personnel, and time limitations may hinder the practice of SSC [53].

The prevalence of TIBF ranged from 36.33% in Gambia to 87.25% in Burundi. Some recent investigations [25, 54] also reported that Burundi recorded the highest prevalence of TIBF in SSA. The difference in sociocultural practices could help explain the current observation among the countries. It could also be that access to health facilities and services in Burundi has relatively improved compared to Gambia [25]. Additionally, compared to Gambia, more women in Burundi could be aware of the benefits of practicing TIBF leading to their higher likelihood of providing TIBF to their infants and young children [25]. Evidence shows that women who deliver in health facilities are more likely to initiate breastfeeding on time [36, 55]. Possibly, most women in Gambia do not give birth at the health facilities, reducing their propensity of practicing TIBF [56].

The importance of breastfeeding for both the mother and the baby during the period of breastfeeding have been documented in literature [7–9]. Evidence has shown that breast milk is the best food for children in the first two years of their life and replacing it with any other thing is not recommended [7]. Additionally, breastfeeding increases child’s innate nutritional behavior including searching and sucking, which is critical for the child’s growth and survival as the mother exclusively feeds them [8, 9]. Breastfeeding also protects infants from diseases and their severity [10–12]. Other studies have indicated that breastfeeding is associated with reduced risk of childhood obesity [13], type 1 diabetes [14, 15], asthma [16], epilepsy [17], including lowering the incidence of breast cancer in mothers [18–20]. Given the importance of breastfeeding, it is surprising to find the low prevalence of SSC and TIBF in SSA.

Similar to the observations of previous studies [35, 41, 57–61], this study found that infants whose mothers practiced SSC were more likely to receive TIBF. A linkage could be drawn to the success in breastfeeding outcomes as a result of the provision of sensory experiences of touch and thermal regulation, which trigger the release of oxytocin for breastmilk production [28, 35]. Improvement in the mothers’ satisfaction and confidence with the initial breastfeeding could have accounted for the observed results in our study [41, 61]. The American College of Nurse-Midwives (ACNM) [62] reiterates that SSC aids infants and young children in identifying and finding their mothers’ nipples by smelling can initiate breastfeeding by themselves. This assertion could be linked to the catecholamine right after child delivery, which intensifies the sensitivity of the child’s nose, particularly the olfactory bulbs [62].

Infants whose mothers practiced SSC and were from Angola, Cameroon, Ethiopia, Guinea, Liberia, Mali, Malawi, Sierra Leone, Tanzania, Uganda, South Africa, Zambia, and Zimbabwe were more likely to receive TIBF. This finding agrees with some previous studies [35, 52]. For example, in Zimbabwe, Mukora-Mutseyekwa et al. [35] found that children who experienced SSC were more likely to also experience TIBF. These countries in SSA may be following the recommendations of WHO on the practice of SSC [63]. Countries in SSA that have low prevalence of SSC and TIBF should take insights from the countries identified.

### Strengths and limitations

Our study is the first to examine the association between mother and newborn SSC and TIBF in SSA. The most recent DHS data from a nationally representative population-based survey was utilized, thereby making observed findings generalizable to mothers and newborns in SSA. Notwithstanding, the cross-sectional design of the DHS limits the analysis, in that we were unable to make causal inferences between SSC and TIBF. Also, the DHS data was collected retrospectively as such, prone to recall bias.

## Conclusions

The prevalence of SCC was relatively low but TIBF was high. Significant inter-country variations of the prevalence of SSC and TIBF were also observed. This study found a strong positive association between SSC and TIBF. To enhance TIBF after birth, more child and maternal healthcare interventions focused on improving mother and newborn SSC should be implemented.
